# Favorable association between Mediterranean diet (MeD) and DASH with NAFLD among Iranian adults of the Amol Cohort Study (AmolCS)

**DOI:** 10.1038/s41598-022-06035-8

**Published:** 2022-02-08

**Authors:** Azam Doustmohammadian, Cain C. T. Clark, Mansooreh Maadi, Nima Motamed, Elham Sobhrakhshankhah, Hossein Ajdarkosh, Mohsen Reza Mansourian, Saeed Esfandyari, Nazanin Asghari Hanjani, Mahsa Nikkhoo, Farhad Zamani

**Affiliations:** 1 Gastrointestinal and Liver Diseases Research Center, Iran University of Medical Sciences, Behafarin St., Karimkhan Ave., Vali-Asr Sq, 1449614535 Tehran, Iran; 2 Centre for Intelligent Healthcare, Coventry University, Coventry, CV1 5FB UK; 3 Department of Social Medicine, Zanjan University of Medical Sciences, Zanjan, Iran; 4 Asadabad School of Medical Sciences, Hamadan, Iran; 5 School of Public Health, Iran University of Medical Sciences, Tehran, Iran

**Keywords:** Diseases, Health care, Risk factors

## Abstract

Nonalcoholic fatty liver disease (NAFLD) is an emerging cause of chronic liver diseases and a major health problem worldwide. Dietary patterns may play a critical role in controlling and preventing this disease, but the available evidence is scarce. The current study aims to ascertain the association of adherence to the Dietary Approach to Stop Hypertension (DASH) diet and Mediterranean diet (MeD) with nonalcoholic fatty liver disease (NAFLD) among Iranian adults of the Amol Cohort Study (AmolCS). In a cross-sectional analysis among 3220 adults (55.3% men), age ≥ 18 years (46.96 ± 14.67), we measured usual dietary intake with a validated food frequency questionnaire (FFQ) and then calculated dietary pattern scores for DASH and MeD. Sociodemographic and lifestyle factors were collected by a structured questionnaire. The presence and degree of NAFLD were also determined by abdominal sonography. Multiple regression models were used to estimate NAFLD odds across tertiles of DASH and Mediterranean dietary scores. Dietary DASH and Mediterranean components were adjusted for total energy intake, based on the residual methods. After adjusting for multiple potential confounders, we found an inverse association of DASH and MeD with NAFLD (Ptrend = 0.02, and Ptrend = 0.002, respectively). Those in the highest tertiles of adherence to the DASH and MeD had the lowest risk for NAFLD (OR = 0.80, 95%CI = 0.66–0.96, OR = 0.64, 95%CI = 0.52–0.78, respectively). The results of logistic analysis of MeD, stratified by gender and abdominal obesity, revealed the favorable association was more pronounced in women (OR = 0.42, 95%CI = 0.29–0.61, Ptrend = 0.004), and in participants with or without abdominal obesity (OR = 0.62, 95% CI = 0.47–0.81, Ptrend = 0.03, OR = 0.64, 95%CI = 0.475–0.91, Ptrend = 0.04, respectively). Similar results were obtained for the adherence to DASH diet score with the prevalence of NAFLD patients with abdominal obesity (OR = 0.75, 95% CI = 0.57–0.97, Ptrend = 0.04). The findings suggested the favorable association between DASH and MeD with NAFLD in Iranian adults, especially women and subjects with or without abdominal obesity. Further prospective investigations are needed to confirm the integrity of our findings.

## Introduction

Nonalcoholic fatty liver disease (NAFLD) is an emerging cause of chronic liver diseases and a major health problem worldwide^1^. Generally, NAFLD is a term used to describe a wide pathological spectrum, ranging from steatosis, steatohepatitis, steatonecrosis, and cirrhosis, resulting from fat accumulation in the liver^2^. Epidemiological data suggest that NAFLD prevalence is about 25%, ranging from 13% in Africa to 42% in Southeast Asia^3^. As a major health problem, NAFLD is a well-known risk factor of cardiovascular diseases and the leading cause of liver transplantation in men and the second in women^4^.

To date, researchers conducted an intensive study to understand NAFLD etiology better and seek the most effective dietary interventions for treatment. Given there is no consensus with respect to the pharmacological treatment of NAFLD, most therapeutic approaches focus on calorie restriction and physical activity to reach a gradual weight decline. For these reasons, dietary interventions remain the cornerstone of NAFLD management. Similarly, considering the role of diet composition to improve metabolic function is gaining increased attention^5,6^.

Food groups, food items, and individual nutrients are the components of dietary patterns and represent one of the key lifestyle factors involved in controlling and preventing NAFLD^7^. The Dietary Approaches to Stop Hypertension (DASH) diet is a healthful low-glycemic index and low-energy-dense diet initially designed to reduce hypertension^8,9^. This dietary pattern includes a wide variety of high-quality foods rich in antioxidants, magnesium, potassium, and dietary fiber and discourages red or processed meat, sugar, and sodium intakes^10,11^. In addition to antihypertensive effects, DASH diet is an effective approach for improving chronic diseases, including cardiovascular risks, diabetes, and metabolic syndrome^12^. Shirani et al., in a meta-analysis, reported that DASH diet score is associated with insulin sensitivity improvement, and some other studies posited that inflammatory markers reduction might be seen as a result of DASH diet adherence^13^.

The Mediterranean diet (MeD) is defined as a plant-based diet characterized by a high intake of fruits and vegetables, legumes, whole grains, and a high ratio of monounsaturated fatty acids (MUFA), which is associated with a lower risk of many chronic diseases^2^. Studies have suggested favorable health outcomes followed by adherence to this dietary pattern, including reducing NAFLD severity^14^. The EASL-EASD-EASO Clinical Practice Guidelines have recently recommended MeD as a dietary choice for NAFLD treatment, particularly through a reduction in insulin resistance and lipid serum concentrations. It can induce regression of steatosis, a significant reduction of cardiovascular events^15^. Despite the apparent benefits of the Mediterranean and DASH diet for Type 2 diabetes and cardiovascular disease^16,17^, the evidence for their efficacy in NAFLD is limited^18^.

Currently, the exact effects and composition of dietary patterns have not been clearly established, and most studies were conducted in Western populations. Indeed, different components of these two dietary patterns in Iranian people and Middle Eastern differ from Western countries^19,20^. Therefore, owing to the distinct lack of large-scale evidence from an Iranian population, the current study investigates the association of MeD and DASH patterns with NAFLD in a large-scale sample of Iranian adults who participated in the Amol Cohort Study (AmolCS).

## Results

### General characteristics of the study participants

Of the 3220 recruited in the cohort, 1437 (44.6%) had NAFLD, and 1438 (44.7%) were women. The mean age of participants was 46.96 ± 14.67 years, and all baseline characteristics are shown in Table 1. Anthropometry and metabolic traits were significantly different in NAFLD and non-NAFLD patients. Compared with men, women had a higher rate of BMI (body mass index), diabetes, metabolic syndrome, the use of lipid-lowering agents and hypertension-lowering agents, urban residency, total cholesterol, HDL, FBS, HbA1C, and a lower rate of waist circumstance, alcohol consumption, smoking, heart disease, physical activity level, triglyceride, AlT, AST, GGT (all p < 0.05).

**Table 1:** Baseline characteristics of the study adult participants (n = 3220, aged ≥ 18 years), Amol Cohort Study, Iran, 2016–2017.

Characteristic	Women		Men		P value	Non-NAFLD		NAFLD		P value
	N/mean	%/SD	N/mean	%/SD		N/mean	%/SD	N/mean	%/SD	
n and %	1438	44.7	1782	55.3	–	1783	55.4	1437	44.6	–
Age (years)	45.68	14.05	48.00	15.08	< 0.001	45.59	16.11	48.65	12.47	< 0.001
BMI (kg/m^2^)	29.52	5.33	26.78	4.34	< 0.001	25.96	4.38	30.54	4.53	< 0.001
Waist circumference (cm)	87.90	12.05	89.63	10.48	< 0.001	83.91	9.95	94.95	9.64	< 0.001
Smoker	8	0.6	459	25.8	< 0.001	285	16.00	182	12.70	0.008
Alcohol drinker	5	0.40	233	10.70	< 0.001	97	5.60	92	6.60	0.24
Diabetes	278	19.30	196	11.00	< 0.001	174	9.80	300	20.09	< 0.001
Metabolic syndrome	512	35.60	354	19.90	< 0.001	238	13.40	628	43.70	< 0.001
Heart disease	50	3.50	94	5.30	0.01	81	4.50	63	4.40	0.82
Lowering serum glucose agent’s user (%)	90	6.3	94	5.3	0.23	73	4.1	111	7.9	< 0.001
Lowering serum lipid agent’s user (%)	204	14.2	175	9.8	< 0.001	181	10.2	198	13.8	0.001
Lowering hypertension agent’s user (%)	303	21.1	290	16.3	< 0.001	288	16.2	305	21.2	< 0.001
**Residual areas**										
Rural	520	36.20	905	50.08	< 0.001	788	44.20	637	44.30	0.94
Urban	918	63.80	877	49.20		995	55.80	800	55.70	
**PA (MET-h/d)**										
Very low	486	34.20	506	28.70	< 0.001	514	29.30	478	33.50	0.06
Low	582	41.0	676	38.30		703	40.0	555	38.90	
Moderate	72	5.10	101	5.70		102	5.80	71	5.0	
high	280	19.70	482	27.30		438	24.90	324	22.70	
TG (mg/dl)	127.93	88.34	138.15	91.23	0.001	111.79	65.41	160.63	107.50	< 0.001
Total cholesterol (mg/dl)	183.47	41.94	178.31	38.85	< 0.001	176.12	39.16	186.19	41.08	< 0.001
HDL(mg/dl)	46.10	11.76	41.77	11.36	< 0.001	44.70	11.81	42.48	11.52	< 0.001
LDL(mg/dl)	99.69	26.72	98.72	26.16	0.30	97.45	26.65	101.26	25.97	< 0.001
SBP (mmHg)	113.51	20.48	115.79	18.04	0.001	111.24	18.30	119.15	19.40	< 0.001
DBP (mmHg)	70.78	12.28	72.36	11.36	< 0.001	69.14	11.11	74.78	11.89	< 0.001
FBS (mg/dl)	108.45	40.33	103.68	29.79	< 0.001	100.85	30.77	111.97	38.70	< 0.001
HbA1C	4.60	0.94	4.53	0.90	0.02	4.46	0.86	4.69	0.96	< 0.001
ALT (mg/dl)	19.74	13.89	27.50	20.23	< 0.001	20.19	14.60	28.81	20.70	< 0.001
AST (mg/dl)	19.44	8.22	23.35	11.56	< 0.001	20.73	10.89	22.69	9.62	< 0.001
GGT (mg/dl)	24.04	18.54	29.42	19.07	< 0.001	23.86	17.79	30.94	19.76	< 0.001
ALKP (mg/dl)	195.87	67.37	199.34	53.05	0.10	193.08	61.76	203.65	56.96	< 0.001

NAFLD: nonalcoholic fatty liver disease; PA: physical activity, MET: metabolic equivalent of task, BMI: body mass index, ALT: alanine transaminase, AST: aspartate transaminase, GGT: gamma-glutamyl transferase, ALKP: alkaline phosphatase.

Significant at P < 0.05 for independent t-test for continuous variables and chi-square test for dichotomous variables.

The Characteristics of the study population across tertiles of DASH and MeD scores are provided in Table 2. Total energy intake was higher in the men compared to the women (2458 vs. 2171 kcal/day) (p < 0.05). After residual adjustment for energy intake, compared with men, women had higher DASH and lower Mediterranean diet scores (p < 0.05). According to the DASH component, women had a higher intake of vegetables and lower dairy products but lower red and processed meat and sweetened beverages (p < 0.05). According to the Mediterranean diet component, women had a higher intake of fruit and vegetables and a lower intake of poultry, total meat, and red meat. Also, non-NAFLD patients had a higher Mediterranean diet score compared to NAFLD cases (p < 0.05). Dietary component intakes of the participants across tertiles of dietary DASH score are shown in Supplementary Tables S1 and S2 online.

**Table 2:** Dietary patterns of the study adult participants (n 3220, aged ≥ 18 years), Amol Cohort Study, Iran, 2016–2017.

Characteristic	Women		Men		P value	Non-NAFLD		NAFLD		P value
	N/mean	%/SD	N/mean	%/SD		N/mean	%/SD	N/mean	%/SD	
**Components of DASH diet**										
Energy (kcal/d)	2171.07	599.92	2458.24	685.51	< 0.001	2326.48	668.73	2334.35	658.55	0.73
Vegetables (serving/d)	2.73	1.67	2.60	1.88	0.04	2.61	1.78	2.72	1.79	0.08
Fruits (serving/d)	2.82	3.56	3.01	4.42	0.20	2.99	4.17	2.85	3.91	0.34
Nuts and legumes (serving /d)	1.75	1.21	1.69	1.33	0.18	1.71	1.30	1.72	1.25	0.93
Low Dairy products (serving/d)	0.56	0.43	0.53	0.47	0.04	0.54	0.47	0.54	0.44	0.97
Whole grains and grain products (serving/d)	5.06	4.73	4.74	5.27	0.07	4.88	5.08	4.89	4.99	0.96
Red and processed meats (serving/d)	0.15	0.25	0.18	0.36	0.03	0.16	0.27	0.17	0.37	0.56
Sweetened beverages (ml/d)	0.01	0.12	0.02	0.18	0.005	0.02	0.14	0.02	0.17	0.87
Salt (g/d)	2499.61	1624.83	2451.50	2118.69	0.47	2463.82	1952.80	2484.36	1864.94	0.76
DASH score	27.88	2.93	27.44	3.24	< 0.001	27.67	3.18	27.59	3.03	0.50
**Components of MeD**										
Vegetables (g/d)	311.82	183.88	297.39	200.62	0.03	298.35	186.79	310.64	201.22	0.07
Non-refined cereals	145.83	137.73	136.11	153.93	0.06	140.30	147.99	140.65	145.76	0.94
Total cereal (g/d)	305.25	143.43	308.81	160.81	0.51	309.07	155.79	304.93	150.12	0.44
Fermented dairy (g/d)	454.47	139.95	455.03	140.51	0.91	451.21	138.84	459.20	141.87	0.10
Legume, nut and seed (g/d)	41.78	57.26	43.10	77.73	0.59	43.05	67.73	41.84	71.28	0.62
Total dairy (g/d)	453.33	311.28	452.23	328.80	0.92	452.16	324.26	453.41	317.11	0.91
Low fat dairy (g/d)	84.02	151.02	87.02	163.49	0.59	83.13	154.52	88.84	162.27	0.30
Red meat (g/d)	15.28	16.61	18.77	26.32	< 0.001	16.69	17.95	17.85	27.23	0.14
Process meat (g/d)	2.62	7.58	2.97	8.09	0.21	3.01	8.58	2.57	6.88	0.11
Poultry (g/d)	70.54	76.37	76.79	94.39	0.04	71.08	87.19	77.61	86.33	0.03
Fish (g/d)	2.16	21.41	3.53	32.81	0.17	3.11	29.43	2.68	26.83	0.66
Total meat (g/d)	87.67	79.42	96.02	101.69	0.01	89.08	91.20	96.27	93.94	0.02
MUFA/SFA (g/d)	0.89	0.30	0.89	0.33	0.70	0.91	0.33	0.88	0.29	0.005
Fruits (g/d)	288.48	213.12	304.81	222.66	0.03	299.77	222.16	294.72	214.08	0.51
Oleic/SFA (g/d)	0.69	0.23	0.69	0.23	0.65	0.71	0.24	0.67	0.22	< 0.001
Med diet score	3.20	1.32	3.51	1.41	< 0.001	3.44	1.41	3.29	1.33	0.002

NAFLD: nonalcoholic fatty liver disease; DASH: Dietary Approaches to Stop Hypertension, MeD: Mediterranean diet; MUFA: monounsaturated fatty acids; SFA: saturated fatty acid.

Significant at P < 0.05 for Independent t-test for continuous variables.

### Association among dietary patterns and other factors with NAFLD

The risk of NAFLD (odds and 95% CI) in each tertile of DASH, as well as Mediterranean diet, score is shown in Table 3. In model 3, after adjusting for multiple potential confounders, we found an inverse association of adherence to DASH and Mediterranean diet with odds of NAFLD (OR = 0.80, 95% CI = 0.66–0.96, p for trend = 0.02; OR = 0.64, 95% CI = 0.52–0.78, p for trend = 0.002, respectively). After stratification for sex, these inverse associations of DASH and Mediterranean diet remained significant for women (OR = 0.72, 95% CI = 0.53–0.98, p for trend = 0.05; OR = 0.42, 95% CI = 0.29–0.61, p for trend = 0.004, respectively).

**Table 3:** Multivariable-adjusted odds ratio and 95% confidence intervals for nonalcoholic fatty liver disease (NAFLD) according to tertiles of dietary patterns in all adult participants and stratified by sex, Amol Cohort Study, Iran, 2016–2017 (n 3220).

	DASH				MeD			
	Tertile 1	Tertile 2	Tertile 3	P_trend_	Tertile 1	Tertile 2	Tertile 3	P_trend_
**NAFLD compared with non-NAFLD**								
Median score	25	28	31		2	4	5	
NAFLD cases (N, %)	1386 (43)	981 (30.5)	853 (26.5)		1675 (52)	886 (27.5)	659 (20.5)	
Model 1	Ref	0.99 (CI 0.84–1.17)	0.81 (CI 0.68–0.96)	0.02	Ref	1.01 (CI 0.75–1.36)	0.63 (CI 0.45–0.90)	0.003
Model 2	Ref	1.00 (CI 0.84–1.18)	0.79 (CI 0.66–0.94)	0.01	Ref	1.11 (CI 0.94–1.32)	0.65 (CI 0.53–0.79)	0.005
Model 3	Ref	1.02 (CI 0.85–1.21)	0.80 (CI 0.66–0.96)	0.02	Ref	1.07 (CI 0.90–1.27)	0.64 (CI 0.52–0.78)	0.002
**Women**								
Median score	26	28	31		2	4	5	
NAFLD cases (N, %)	570 (39.6)	469 (32.6)	399 (27.7)		820 (57)	392 (27.3)	226 (15.7)	
Model 1	Ref	0.95 (CI 0.74–1.23)	0.73 (CI 0.56–0.95)	0.02	Ref	1.15 (CI 0.90–1.48)	0.65 (CI 0.33–0.65)	0.006
Model 2	Ref	1.02 (CI 0.76–1.36)	0.71 (CI 0.52–0.97)	0.04	Ref	1.13 (CI 0.85–1.50)	0.43 (CI 0.30–0.63)	0.006
Model 3	Ref	1.02 (CI 0.76–1.36)	0.72 (CI 0.53–0.98)	0.05	Ref	1.10 (CI 0.83–1.47)	0.42 (CI 0.29–0.61)	0.004
**Men**								
Median score	25	29	31		2	4	5	
NAFLD cases (N, %)	816 (45.8)	512 (28.7)	454 (25.5)		855 (48)	494 (27.7)	433 (24.3)	
Model 1	Ref	1.04 (CI 0.83–1.30)	0.93 (CI 0.74–1.18)	0.64	Ref	1.04 (CI 0.83–1.30)	0.77 (CI 0.61–0.98)	0.12
Model 2	Ref	1.03 (CI 0.78–1.34)	0.89 (CI 0.67–1.19)	0.52	Ref	1.11 (CI 0.85–1.45)	0.72 (CI 0.54–0.95)	0.12
Model 3	Ref	1.01 (CI 0.77–1.33)	0.88 (CI 0.66–1.17)	0.45	Ref	1.06 (CI 0.81–1.40)	0.70 (CI 0.53–0.94)	0.07

DASH: Dietary Approaches to Stop Hypertension, MeD: Mediterranean diet, Ref: reference category.

Model 1: adjusted for age and sex.

Model 2: Additional adjustment for BMI, energy intake, physical activity, and smoking.

Model 3: additional adjustment for Lowering serum lipid drugs, Lowering HPTN drugs, Lowering serum glucose drugs, residual areas, heart disease, diabetes.

Table 4 presents the association of NAFLD risk with DASH and Mediterranean diet score stratified by abdominal and non-abdominal obesity. In model 3, after adjusting for all confounders, there was an inverse association between adherence to DASH diet score with the prevalence of NAFLD patients with abdominal obesity (OR = 0.75, 95% CI = 0.57–0.97, p for trend = 0.04). Similar results were obtained for Mediterranean diet score and prevalence NAFLD patients with or without abdominal obesity (OR = 0.62, 95% CI = 0.47–0.81, p for trend = 0.03; OR = 0.64, 95% CI = 0.475–0.91, p for trend = 0.04 respectively).

**Table 4:** Multivariable-adjusted odds ratio and 95% confidence intervals for nonalcoholic fatty liver disease (NAFLD) according to tertiles (T) of dietary patterns in all adult participants stratified by waist circumference, Amol Cohort Study, Iran, 2016–2017 (n = 3220).

	DASH				MeD			
	Tertile 1	Tertile 2	Tertile 3	P_trend_	Tertile 1	Tertile 2	Tertile 3	P_trend_
**NAFLD compared with non-NAFLD** **Non_abdominal obesity**								
NAFLD cases (N, %)	134 (43.8)	97 (31.7)	75 (24.5)		166 (54.2)	88 (28.8)	52 (17)	
Model 1	Ref	1.07 (CI 0.80–1.45)	0.89 (CI 0.65–1.23)	0.57	Ref	1.01 (CI 0.75–1.36)	0.63 (CI 0.45–0.90)	0.05
Model 2	Ref	1.05 (CI 0.78–1.43)	0.85 (CI 0.61–1.18)	0.39	Ref	0.98 (CI 0.72–1.33)	0.64 (CI 0.45–0.92)	0.05
Model 3	Ref	1.03 (CI 0.76–1.40)	0.87 (CI 0.63–1.21)	0.40	Ref	0.96 (CI 0.71–1.31)	0.64 (CI 0.45–0.91)	0.04
**NAFLD compared with non-NAFLD** **Abdominal obesity**								
Abdominal obesity^a^ cases	497 (43.9)	357 (31.6)	277 (24.5)		600 (53.1)	343 (30.3)	188 (16.6)	
Model 1	Ref	0.95 (CI 0.75–1.21)	0.76 (CI 0.59–0.96)	0.03	Ref	1.16 (CI 0.92–1.46)	0.65 (CI 0.50–0.84)	0.05
Model 2	Ref	0.98 (CI 0.77–1.25)	0.76 (CI 0.59–0.98)	0.04	Ref	1.21 (CI 0.95–1.55)	0.64 (CI 0.49–0.83)	0.07
Model 3	Ref	0.97 (CI 0.76–1.24)	0.75 (CI 0.57–0.97)	0.04	Ref	1.17 (CI 0.92–1.50)	0.62 (CI 0.47–0.81)	0.03

DASH: dietary approaches to stop hypertension, MeD: Mediterranean diet, Ref: reference category.

Model 1: adjusted for age and sex.

Model 2: additional adjustment for BMI, energy intake, physical activity, and smoking.

Model 3: additional adjustment for Lowering serum lipid drugs, Lowering HPTN drugs, Lowering serum glucose drugs, residual areas, heart disease, diabetes.

^a^Abdominal obesity: waist circumference > 102 cm for men and > 88 cm for women.

## Discussion

The current study investigated the association of a priori defined DASH and MeD dietary patterns with NAFLD. This study contributes to furthering our understanding of the associations between diet and diseases in specific world regions, where social factors and dietary patterns may be distinctive. Indeed, the findings suggested that favorable adherence to healthful dietary patterns, including DASH and MeD, was inversely associated with NAFLD risk in Iranian adults.

Our findings are consistent with a cross-sectional analysis of a cohort study on 3051 Chinese adults aged 40–75 years, which revealed adherence to the DASH diet was independently associated with a lower prevalence of NAFLD^21^. A previous randomized controlled clinical trial in 60 overweight or obese Iranian adults with NAFLD revealed adherence to the DASH diet for 8 weeks, compared with the control diet, improved liver enzymes, and some NAFLD risk factors, including weight and serum triglycerides, and inflammatory and insulin resistance markers^22^.

In addition, the inverse association MeD on NAFLD in our study was concordant with other studies^4,23,24^. For example, a cohort study by Baratta et al. showed that adherence to MeD had a preventive effect on NAFLD (OR: 0.801, P = 0.018)^25^. Further, a meta-analysis including seven observational reports and six randomized clinical trials showed that adherence of MeD can significantly reduce BMI (effect size = − 1.23 kg/m^2^; 95% CI − 2.38 to − 0.09 kg/m^2^), weight (effect size =  − 4.13 kg; 95% CI − 8.06 to − 0.20 kg), serum triglycerides (effect size =  − 33.01 mg/dL; 95% CI − 52.84 to − 13.18 mg/dL), and total cholesterol (effect size =  − 6.89 mg/dL; 95% CI − 14.90 to 1.12 mg/dL)^26^, which can theoretically translate to protective effects in NAFLD. Moreover, Kaliora et al., in a 24-week prospective diet intervention study on 44 NAFLD patients with simple steatosis, concluded that adherence to MeD improves liver imaging, liver fibrosis score, blood pressure, fasting glucose, HgA1C, and several other biomarkers, compared with pre-intervention values^27^.

Several mechanisms have been posited to contribute to the desirable effects of adherence to healthful dietary patterns, including MeD and DASH, on NAFLD, which consist mainly of higher intake of vegetables, fruits, plant-based proteins (nuts and legumes), whole-grain cereals, and micronutrients such as calcium, potassium, and vitamin C, dietary fiber, monounsaturated fatty acids, and omega-3 fatty acids, as well as polyphenols and other antioxidant agents^28^. Currently, strong evidence-based data supports the advantages of healthy dietary patterns in controlling most risk factors for NAFLD^16^; for instance, Dorosty et al. reported that consumption of whole grains for 12 weeks, independent of weight loss, beneficially affected liver enzymes concentrations, and fatty liver in patients with NAFLD^29^. Based on the observed benefits of the MeD components on NAFLD in our study, non-NAFLD individuals had a greater intake of MUFA/SFA and lower intake of animal protein sources (red meats and poultry) than those with NAFLD. Current evidence-based data also suggests a positive association between consumption of red meats and chicken, rather than marine animals, with hepatic status in obese subjects^30^.

Regarding the possible mechanism associated with a MeD and NAFLD, its effect on improving cardiometabolic outcomes (such as reducing triglycerides or fasting plasma glucose)^31^ or the antioxidant properties^32^ of healthy diets can be pointed to. On the other hand, the American Heart Association recommendations, which emphasize healthy dietary patterns such as the DASH or MeD, rather than specific food items, can be referred to^33^.

According to our findings, the favorable association of DASH and MeD was more pronounced in women and especially subjects with abdominal obesity. After adjusting for multiple confounders in subjects without abdominal obesity, adherence to MeD was also inversely related to NAFLD. The rationale for why the relationship was significant only among women could be attributed to an increased willingness to adopt a healthy diet. In our study, female participants, compared to males, independent of energy intake, had a greater consumption of fruits, vegetables, and lower dairy products, animal protein, and sweetened beverages.

In the current study, adherence to DASH and the Mediterranean diet was inversely associated with NAFLD prevalence in patients with abdominal obesity. Indeed, a similar result remained for MeD and NAFLD patients without abdominal obesity as well. This result suggests that MeD might represent a better choice for NAFLD management, as reported by evidence^26^.

Although we present a novel addition to the literature, the present study has some limitations. The main limitation of this study is the lack of a liver biopsy as the gold standard to assess the extent of liver damage in NAFLD, which could not be performed on the outpatients, as well as the cross-sectional nature of the study which precludes causal inferences being made. Moreover, recall bias and measurement error related to assessing food intake using validated FFQ cannot be easily ruled out in our study. Further, possible residual effects due to incomplete adjustment for remaining confounders may have affected the outcome variables. Furthermore, since some of the NAFLD participants may have already adopted healthy lifestyles, following physician advice may lead to achieving a high dietary pattern score among patients. However, this tendency may contribute to the unfavorable association between healthy dietary patterns and NAFLD, and we still observed an inverse relationship in our study. Finally, our study population was comprised solely of adults of the north of Iran; therefore, the extrapolation of our findings to all Iranian adults must be made cautiously.

### Conclusion

The findings of our study suggested a favorable association between the adherence to DASH and MeD with NAFLD in Iranian adults, especially women and subjects with or without abdominal obesity. However, further prospective investigations are needed to confirm the veracity of this suggestion.

## Methods

### Study population

This is a population-based cross-sectional study, and participants were drawn from the second phase of AmolCS. A comprehensive data collection was done in two different phases, including 2009–2010 (phase 1) and 2016–2017 (phase 2). A multistage sampling technique was applied. All twenty-five rural and sixteen urban health centers of Amol city, north of Iran, were the source of the sampling frame of the study. Sixteen strata with 10 years intervals including 10–19, 20–29, 30–39, 40–49, 50–59, 60–69, 70–79, 80–89 years of age were selected. The random selection of study subjects in each stratum was conducted proportionally to the population size. Participants with excessive alcohol consumption of more than 30 g/d for men and more than 20 g/d for women, viral hepatitis, and those on medication known to cause fatty liver, weight loss, or weight gain in the last 3 months before sonography (n 486) and pregnancy/lactation (n 153) were excluded.

Laboratory, anthropometric, and demographic variables were comprehensively were collected in each phase^26^. An informed consent form was obtained from all participants before the study, and the study protocol was approved by the Iran University of Medical Sciences ethics committee.

**Figure 1:**
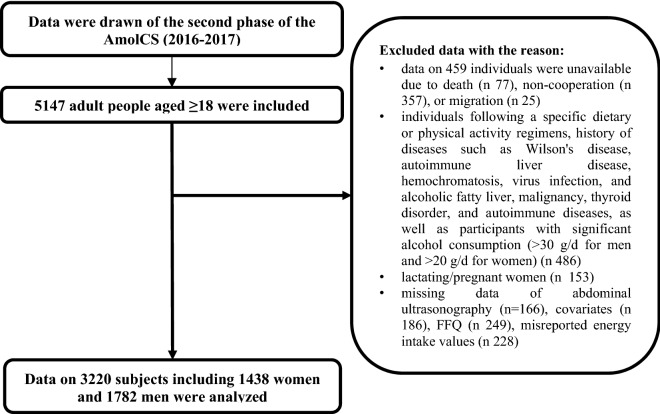
Flowchart of the study design.

After excluding subjects with missing data for abdominal ultrasonography (n 166), covariates (n 186), and food frequency questionnaire (n 249), as well as misreporting energy intake (n 228), ultimately, 3220 subjects, including 1438 women and 1782 men, were analyzed in the study (Fig. 1). Excluded subjects had a comparable socioeconomic level as those who remained in the study.

### Dietary assessment

Recruited patients answered a 168-items semi-quantitative food frequency questionnaire adapted to the Iranian society to assess usual dietary intake^34^. Participants reported their average frequency of each food item on the previous year per day, week, month, and year, or never. The reported portion size and frequency of food intake were converted to daily intake, and the portion size was converted to grams by household measures^35^. Nutrient and energy intake were calculated by the US Department of Agriculture (USDA) Food Composition Table (FCT)^36^ because the Iranian FCT is incomplete. The Iranian FCT was used alternatively for traditional foods not listed in the USDA FCT^37^.

### Creation of dietary scores

We followed the methodology of Fung et al. to calculate DASH dietary scores^38^. Accordingly, we constructed a DASH score based on eight components emphasized or minimized in the DASH diet, and each of them was placed into quintiles. One point was allocated for receiving fruits, vegetables, low-fat dairy products, whole grains, nuts, and legumes at the highest quintile, and for the remaining components (intake of sodium, soft drink or sweet beverage, red or processed meats), low intakes were desirable.

MeD Score was calculated based on the Trichopoulou et al. methodology by taking into account the consumption of nine food groups. Whether the participants' adherence to each MeD component they received a score of 0 or 1^39^. Daily servings of fruits, vegetables, whole grain, nuts, legumes ratio of grams of MUFA to saturated fatty acids (SFA) equivalent to, or greater than, the median intake of the study population, and also daily servings of dairy products and meat poultry, red and processed) less than the median intake of the study population received one point.

Residual method energy adjustment was conducted for all food groups before the score ranking. Finally, participants were categorized into tertiles based on their MeD scores.

### Abdominal ultrasonography

Sonography was used for NAFLD, and it was defined as hepatic steatosis after the exclusion of secondary causes of fat build-up in the liver (e.g., drug-related steatosis, excess alcohol consumption, and viral hepatitis). One expert sonographer performed all ultrasound examinations and was blind and not involved in any of the cohort procedures. Sagittal, longitudinal, lateral, and intercostal views were provided through a 3–5 MHz transducer. Similarly, related criteria for fatty liver confirmation included blurring of portal or hepatic veins plus a marked increase in hepatic echogenicity.

### Laboratory and anthropometric assessments

Serum collected from 2016 to 2017 was applied to the measurement of all laboratory tests. Blood samples were drawn after overnight fasting, and serum obtained from whole blood was used for biochemical analyses using an automatic BS-200 chemistry analyzer (Mindray, China). After whole blood samples incubation at room temperature, they were centrifuged at 3000 rpm for 10 min. Laboratory parameters included; fasting blood sugar (FBS), HDL, TG, total cholesterol, liver enzymes (alanine aminotransferase, aspartate aminotransferase, gamma-glutamyl transferase), CRP, hepatitis B virus surface antigens, and hepatitis C virus antibodies.

After 5 min in a quiet place, participants' blood pressure was measured in a seated position, using a manual sphygmomanometer. Properly fitted cuffs and calibrated monitors were used. Korotkoff noise appearance and disappearance were considered as systolic and diastolic values, respectively. The mean value of the two measurements was taken. The seventh report of the Joint National Committee on Prevention, Detection, Evaluation, and Treatment of High Blood Pressure was used for hypertension evaluation^40^. Weight and height were measured with participants in minimal clothing, and waist circumference (WC) was also measured based on WHO protocols^41^.

### Statistical analysis

Data were presented as means and standard deviations for continuous variables and frequencies and percentages for categorical ones. The Kolmogorov–Smirnov test and histogram were applied to ensure the normal distribution of variables. Differences between qualitative variables were evaluated by the Chi-square test. Independent t-test and one-way analysis of variance (ANOVA) were applied to determine differences between continuous variables in two and more than two groups, respectively. A univariate analysis was performed for potential confounding variables; variables with PE < 0.2 in the univariate analyses were selected for the final multivariable models; PE (P-value for entry) determines which variables should be included in the multivariable model. Statistical analysis was performed using SPSS 24, and results were, a priori, considered significant at P values < 0.05.

We used multiple regression models to estimate NAFLD risk across tertiles of DASH and Mediterranean dietary scores. The associations of DASH and Mediterranean dietary patterns with NAFLD were adjusted for sex, age, BMI, energy intake, smoking status, physical activity, lowering serum lipid drugs, lowering HPTN drugs, lowering serum glucose drugs, residual areas, heart disease, and diabetes. Dietary DASH and Mediterranean components were adjusted for total energy intake, based on the residual methods (22), such that participants' dietary DASH and Mediterranean scores were residually adjusted for energy intake. A linear trend test was performed, considering each ordinal score variable as a continuous variable in the model. To assess the overall trends of odds ratios of NAFLD across tertiles of dietary DASH and Mediterranean diet score, the median of each tertile was used as a continuous variable in the logistic regression models.

### Ethical standards disclosure

The current study was conducted according to the guidelines laid down in the Declaration of Helsinki and procedures involving human subjects/patients were approved by the Iran University of Medical Sciences (IUMS) ethical committee (No.IR.IUMS.REC.1399.1393). Written informed consent was obtained from all participants prior the study.

## Supplementary Information

Supplementary Tables.

## Data Availability

The datasets used and/or analyzed during the current study are available from the corresponding author on reasonable request.
