# Supplementary material for: Favorable association between Mediterranean diet (MeD) and DASH with NAFLD among Iranian adults of the Amol Cohort Study (AmolCS)
Source: Sci Rep. 2022 Feb 8;12:2131. doi: 10.1038/s41598-022-06035-8 (PMC8825797; doi:10.1038/s41598-022-06035-8)
Supplement: Supplementary file 1 — Supplementary Tables. [file 41598_2022_6035_MOESM1_ESM.docx]

Supplementary Info:

**Favorable association between Mediterranean Diet (MeD) and DASH with NAFLD among Iranian adults of the Amol Cohort Study (AmolCS)**

**Running title: Dietary patterns and NAFLD**

Azam Doustmohammadian^1^, Cain C. T. Clark**^2^**, Mansooreh Maadi^1^, Nima Motamed^3^, Elham Sobhrakhshankhah^1^, Hossein Ajdarkosh^1^, Mohsen Reza Mansourian^1^, Saeed Esfandyari ^4^, Nazanin Asghari Hanjani^5^, Mahsa Nikkhoo^1^, Farhad Zamani^1^*

^1^ Gastrointestinal and Liver Diseases Research Center, Iran University of Medical Sciences, Tehran, Iran

^2^Centre for Intelligent Healthcare, Coventry University, Coventry, CV1 5FB, U.K

^3^ Department of Social Medicine, Zanjan University of Medical Sciences, Zanjan, Iran

^4^Asadabad School of Medical Sciences, Hamadan, Iran

^5^ School of Public Health, Iran University of Medical Sciences, Tehran, Iran

**Table 1. Characteristics of adherence among tertiles of dietary patterns Amol Cohort Study, Iran, 2016–2017(n 3220)**

| **Variables** | **DASH Diet** | | | | **MeD** | | | |
| --- | --- | --- | --- | --- | --- | --- | --- | --- |
|  | **Tertile 1** | **Tertile 2** | **Tertile 3** | **P value** | **Tertile 1** | **Tertile 2** | **Tertile 3** | **P value** |
| **N.Total (%)** | 1386(43) | 981(30.5) | 853(26.5) |  | 1675(52) | 886(27.5) | 659(20.5) |  |
| **Age (years)** | 45.81 ± 15.39 | 47.87 ± 14.17 | 47.98 ± 13.74 | < 0.001 | 46.06 ± 14.97 | 47.51 ± 14.31 | 48.52 ± 14.24 | 0.001 |
| **BMI (kg/m2)** | 27.96 ± 5.05 | 28.00 ± 4.97 | 28.08 ± 4.93 | 0.86 | 28.08 ± 5.02 | 28.20 ± 5.18 | 27.54 ± 4.66 | 0.02 |
| **Waist circumference (cm)** | 88.67 ± 11.56 | 89.14 ± 10.83 | 88.74 ± 11.14 | 0.58 | 88.62 ± 11.29 | 89.32 ± 11.33 | 88.71 ± 10.97 | 0.31 |
| **Smoker** | 6.86 | 4.84 | 2.79 | 0.05 | 6.83 | 4.25 | 3.41 | 0.05 |
| **Alcohol drinker** | 3.19 | 1.67 | 1.22 | 0.12 | 3.12 | 1.57 | 1.38 | 0.71 |
| **Lowering serum glucose agents** | 4.90 | 6.00 | 7.30 | 0.05 | 5.50 | 6.20 | 6.0 | 0.75 |
| **Lowering serum lipid agents** | 10.20 | 11.40 | 15.20 | 0.002 | 10.90 | 12.20 | 13.50 | 0.18 |
| **Lowering HTN agents** | 17.70 | 18.50 | 19.80 | 0.47 | 18.10 | 18.60 | 18.80 | 0.91 |
| **Residual areas** |  |  |  |  |  |  |  |  |
| Rural | 22.36 | 12.51 | 9.37 | < 0.001 | 23.01 | 11.55 | 9.68 | 0.11 |
| Urban | 22.98 | 18.44 | 14.31 |  | 29.00 | 15.96 | 10.77 |  |
| **PA (MET-h/d)** |  |  |  |  |  |  |  |  |
| Very low | 47.0 | 25.80 | 27.30 | 0.01 | 54.50 | 25.50 | 26.40 | 0.65 |
| Low | 41.0 | 35.30 | 23.60 |  | 51.60 | 27.40 | 21.0 |  |
| Moderate | 46.40 | 26.20 | 27.40 |  | 45.20 | 28.60 | 26.20 |  |
| high | 43. 70 | 25.90 | 30.50 |  | 49.30 | 27.20 | 23.50 |  |
| **TG (mg/dl)** | 131.73 ± 87.81 | 135.89 ± 83.16 | 134.11 ± 102.27 | 0.52 | 133.69 ± 91.83 | 133.40 ± 90.71 | 133.55 ± 84.71 | 0.99 |
| **Total Cholesterol(mg/dl)** | 179.64 ± 39.07 | 182.35 ± 42.56 | 180.23 ± 39.70 | 0.25 | 180.84 ± 39.74 | 180.05 ± 40.37 | 180.80 ± 41.82 | 0.88 |
| **HDL(mg/dl)** | 43.43 ± 11.16 | 43.72 ± 10.71 | 44.22 ± 13.90 | 0.32 | 43.63 ± 12.09 | 43.85 ± 11.30 | 43.70 ± 11.42 | 0.90 |
| **LDL(mg/dl)** | 98.08 ± 25.52 | 100.65 ± 27.02 | 99.23 ± 27.22 | 0.06 | 98.67 ± 25.68 | 99.88 ± 27.80 | 99.37 ± 26.38 | 0.53 |
| **SBP (mmHg)** | 114.74 ± 19.71 | 115.05 ± 19.00 | 114.64 ± 18.47 | 0.81 | 114.56 ± 19.48 | 115.10 ± 18.96 | 114.87 ± 18.83 | 0.78 |
| **DBP (mmHg)** | 71.82 ± 11.96 |  | 71.55 ± 11.84 | 0.76 | 71.63 ± 11.54 | 71.55 ± 11.89 | 71.87 ± 12.35 | 0.85 |
| **FBS (mg/dl)** | 104.79 ± 32.83 | 107.81 ± 39.13 | 105.15 ± 33.08 | 0.09 | 104.04 ± 32.01 | 108.36 ± 39.78 | 106.89 ± 35.06 | 0.008 |
| **HbA1C (%)** | 4.55 ± 0.90 | 4.62 ± 1.00 | 4.51 ± 0.81 | 0.02 | 4.52 ± 0.89 | 4.64 ± 0.95 | 4.57 ± 0.92 | 0.01 |
| **Metabolic Syndrome** | 11.97 | 8.61 | 6.34 | 0.74 | 14.11 | 7.33 | 5.47 | 0.96 |
| **ALT (mg/dl)** | 23.83 ± 17.13 | 24.52 ± 17.78 | 23.81 ± 20.19 | 0.60 | 23.66 ± 17.19 | 23.37 ± 16.27 | 25.88 ± 22.14 | 0.01 |
| **AST (mg/dl)** | 21.55 ± 9.28 | 22.00 ± 12.21 | 21.18 ± 9.80 | 0.24 | 21.32 ± 9.06 | 21.28 ± 8.89 | 22.75 ± 14.53 | 0.006 |
| **GGT (mg/dl)** | 26.89 ± 18.31 | 27.28 ± 18.59 | 26.90 ± 20.84 | 0.87 | 26.66 ± 18.42 | 27.27 ± 17.97 | 27.56 ± 21.72 | 0.52 |
| **ALKP (mg/dl)** | 197.80 ± 61.01 | 198.86 ± 54.74 | 196.38 ± 64.06 | 0.69 | 197.44 ± 59.21 | 195.43 ± 56.20 | 201.87 ± 65.98 | 0.10 |

DASH: Dietary Approaches to Stop Hypertension, MeD: Mediterranean diet PA: physical activity, MET: metabolic equivalent of task, BMI: body mass index, ALT: Alanine transaminase, AST: Aspartate transaminase, GGT: Gamma-glutamyl transferase, ALKP: Alkaline phosphatase

Signiﬁcant at P < .05 for one way ANOVA for continuous independent variables and chi-square test for dichotomous variables

**Table 2**. **Dietary intakes of adult participants across tertiles of dietary patterns Amol Cohort Study, Iran, 2016–2017(n 3220)**

| **Variables** | **DASH Diet** | | | | **MeD** | | | |
| --- | --- | --- | --- | --- | --- | --- | --- | --- |
|  | **Tertile 1** | **Tertile 2** | **Tertile 3** | **P value** | **Tertile 1** | **Tertile 2** | **Tertile 3** | **P value** |
| **N.Total (%)** | 1386(43) | 981(30.5) | 853(26.5) |  | 1675(52) | 886(27.5) | 659(20.5) |  |
| **Energy (kcal/day)** | 2325.53 ± 667.25 | 2323.33 ± 656.09 | 2347.25 ± 669.08 | 0.71 | 2371.23 ± 660.61 | 2285.18 ± 642.59 | 2285.44 ± 695.01 | 0.001 |
| **Carbohydrate (E %)** | 52.77 ± 7.73 | 53.71 ± 6.75 | 54.33 ± 6.54 | < 0.001 | 52.23 ± 6.70 | 54.40 ± 6.80 | 55.19 ± 8.30 | < 0.001 |
| **Protein (E %)** | 17.48 ± 5.25 | 16.64 ± 3.22 | 16.27 ± 3.15 | < 0.001 | 17.57 ± 3.59 | 16.55 ± 4.55 | 15.84 ± 5.16 | < 0.001 |
| **Fat (E %)** | 33.23 ± 15.97 | 33.19 ± 15.58 | 32.79 ± 5.72 | 0.77 | 32.98 ± 5.91 | 32.86 ± 14.30 | 33.79 ± 24.63 | 0.37 |
| **SFA (g/d)** | 31.00 ± 13.16 | 31.26 ± 11.59 | 31.96 ± 12.35 | 0.22 | 33.57 ± 12.76 | 29.90 ± 11.84 | 27.44 ± 11.45 | < 0.001 |
| **Cholesterol (g/d)** | 345.18 ± 288.96 | 317.15 ± 159.06 | 308.86 ± 132.23 | < 0.001 | 347.58 ± 162.81 | 301.09 ± 172.26 | 313.89 ± 368.03 | < 0.001 |
| **Whole grains (g/d)** | 172.68 ± 174.56 | 184.28 ± 139.99 | 173.17 ± 131.44 | 0.15 | 155.93 ± 157.67 | 180.61 ± 144.19 | 222.72 ± 151.42 | < 0.001 |
| **Vegetables(g/d)** | 262.98 ± 151.56 | 355.98 ± 176.20 | 479.68 ± 221.38 | < 0.001 | 310.11 ± 182.25 | 370.73 ± 196.53 | 389.92 ± 219.90 | < 0.001 |
| **Low fat dairy (g/d)** | 122.69 ± 134.48 | 151.51 ± 166.61 | 183.76 ± 183.44 | < 0.001 | 148.77 ± 159.41 | 150.41 ± 156.14 | 133.46 ± 162.30 | 0.07 |
| **Meat (g/d)** | 18.67 ± 29.53 | 16.66 ± 15.37 | 15.12 ± 12.85 | 0.001 | 16.50 ± 17.60 | 17.77 ± 30.65 | 18.25 ± 20.93 | 0.16 |
| **Nuts (g/d)** | 4.60 ± 7.89 | 5.80 ± 9.95 | 7.67 ± 10.47 | < 0.001 | 4.06 ± 5.67 | 6.52 ± 11.36 | 8.72 ± 12.24 | < 0.001 |
| **Fruits (g/d)** | 276.37 ± 200.79 | 351.99 ± 191.91 | 474.73 ± 246.19 | < 0.001 | 287.45 ± 172.55 | 385.91 ± 247.33 | 445.00 ± 258.17 | < 0.001 |
| **Legumes (g/d)** | 48.13 ± 60.89 | 59.86 ± 80.39 | 61.01 ± 67.13 | < 0.001 | 42.76 ± 55.31 | 63.62 ± 84.11 | 73.65 ± 72.65 | < 0.001 |
| **Poultry (g/d)** | 97.15 ± 85.78 | 92.89 ± 90.01 | 96.21 ± 90.99 | 0.49 | 112.17 ± 89.11 | 85.71 ± 80.56 | 66.81 ± 87.14 | < 0.001 |
| **Fish (g/d)** | 9.58 ± 30.77 | 7.32 ± 32.65 | 6.61 ± 13.47 | 0.03 | 4.39 ± 8.79 | 9.48 ± 41.15 | 16.03 ± 36.82 | < 0.001 |
| **Sodium (mg/d)** | 3128.59 ± 2765.38 | 2658.09 ± 1489.31 | 2512.44 ± 1383.59 | < 0.001 | 2771.70 ± 1250.06 | 2682.12 ± 2015.68 | 3210.75 ± 3643.21 | < 0.001 |
| **Iron (mg/day)** | 24.56 ± 12.77 | 25.00 ± 12.90 | 26.83 ± 15.35 | 0.001 | 24.51 ± 12.43 | 25.84 ± 15.01 | 26.25 ± 13.84 | 0.006 |
| **Magnesium (mg/d)** | 454.90 ± 160.58 | 465.75 ± 159.00 | 469.29 ± 154.62 | 0.08 | 457.48 ± 156.63 | 462.55 ± 151.18 | 471.12 ± 174.57 | 0.17 |
| **Zinc (mg/d)** | 12.69 ± 4.46 | 12.64 ± 4.22 | 12.45 ± 4.16 | 0.45 | 12.84 ± 4.27 | 12.39 ± 4.24 | 12.36 ± 4.52 | 0.01 |
| **Vitamin C (mg/d)** | 120.01 ± 71.49 | 132.31 ± 68.23 | 167.23 ± 87.22 | < 0.001 | 129.37 ± 72.70 | 140.54 ± 78.62 | 141.90 ± 83.51 | < 0.001 |
| **Potassium (mg/d)** | 4040.33 ± 1301.49 | 4206.25 ± 1285.00 | 4606.71 ± 1467.10 | < 0.001 | 4243.86 ± 1313.88 | 4243.57 ± 1360.26 | 4156.56 ± 1451.39 | 0.33 |
| **Calcium (mg/d)** | 1215.10 ± 490.31 | 1260.62 ± 490.15 | 1329.57 ± 532.55 | < 0.001 | 1309.98 ± 506.95 | 1234.03 ± 512.59 | 1149.90 ± 456.75 | < 0.001 |
| **B12 (mcg/d)** | 6.63 ± 7.47 | 6.21 ± 4.74 | 6.17 ± 5.16 | 0.13 | 6.66 ± 5.77 | 5.80 ± 5.28 | 6.49 ± 8.12 | 0.003 |
| **Dietary fiber (g/d)** | 36.83 ± 14.79 | 37.53 ± 14.67 | 39.09 ± 14.72 | 0.003 | 36.30 ± 14.46 | 38.10 ± 14.14 | 40.58 ± 15.93 | < 0.001 |

DASH: Dietary Approaches to Stop Hypertension, MeD: Mediterranean diet; MUFA: monounsaturated fatty acids, SFA: saturated fatty acid

Signiﬁcant at P < .05 for one way ANOVA for continuous independent variables
